# Use of a Pan–Genomic DNA Microarray in Determination of the Phylogenetic Relatedness among *Cronobacter* spp. and Its Use as a Data Mining Tool to Understand *Cronobacter* Biology

**DOI:** 10.3390/microarrays6010006

**Published:** 2017-03-04

**Authors:** Ben D. Tall, Jayanthi Gangiredla, Christopher J. Grim, Isha R. Patel, Scott A. Jackson, Mark K. Mammel, Mahendra H. Kothary, Venugopal Sathyamoorthy, Laurenda Carter, Séamus Fanning, Carol Iversen, Franco Pagotto, Roger Stephan, Angelika Lehner, Jeffery Farber, Qiong Q. Yan, Gopal R. Gopinath

**Affiliations:** 1 Center of Food Safety and Applied Nutrition, U. S. Food and Drug Administration, Laurel, MD 20708, USA; jayanthi.gangiredla@fda.hhs.gov (J.G.); Christopher.grim@fda.hhs.gov (C.J.G.); Isha.patel@fda.hhs.gov (I.R.P.); scott.jackson@nist.gov (S.A.J.); mark.mammel@fda.hhs.gov (M.K.M.); mahendrakothary@gmail.com (M.H.K.); venugopal.sathyamoorthy@fda.hhs.gov (V.S.); laurenda.carter@fda.hhs.gov (L.C.); gopal.gopinathrao@fda.hhs.gov (G.R.G.); 2 Complex Microbial Systems Group Biosystems and Biomaterials Division, National Institute of Standards and Technology, Gaithersburg, MD 20899, USA; 3 UCD Centre for Food Safety, School of Public Health, Physiotherapy & Population Science, University College, Dublin, Belfield, Dublin D04 N2E5, Ireland; sfanning@ucd.ie (S.F.); qyan07@gmail.com (Q.Q.Y.); 4 College of Life Sciences, University of Dundee, Dundee, DD1 5EH Scotland, UK; c.iversen@dundee.ac.uk; 5 Food Directorate, Bureau of Microbial Hazards, Health Canada, Ottawa, ON K1A 0K9, Canada; Franco.Pagotto@hc-sc.gc.ca; 6 Institute for Food Safety and Hygiene, University of Zurich, Winterthurerstr. 272, CH-8057 Zurich, Switzerland; stephanr@fsafety.uzh.ch (R.S.); lehnera@fsafety.uzh.ch (A.L.); 7 Department of Food Science, University of Guelph, Guelph, ON N1G 2W1, Canada; jfarber@uoguelph.ca

**Keywords:** *Cronobacter*, microarray, data mining

## Abstract

*Cronobacter* (previously known as *Enterobacter sakazakii*) is a genus of Gram-negative, facultatively anaerobic, oxidase-negative, catalase-positive, rod-shaped bacteria of the family *Enterobacteriaceae*. These organisms cause a variety of illnesses such as meningitis, necrotizing enterocolitis, and septicemia in neonates and infants, and urinary tract, wound, abscesses or surgical site infections, septicemia, and pneumonia in adults. The total gene content of 379 strains of *Cronobacter* spp. and taxonomically-related isolates was determined using a recently reported DNA microarray. The *Cronobacter* microarray as a genotyping tool gives the global food safety community a rapid method to identify and capture the total genomic content of outbreak isolates for food safety, environmental, and clinical surveillance purposes. It was able to differentiate the seven *Cronobacter* species from one another and from non-*Cronobacter* species. The microarray was also able to cluster strains within each species into well-defined subgroups. These results also support previous studies on the phylogenic separation of species members of the genus and clearly highlight the evolutionary sequence divergence among each species of the genus compared to phylogenetically-related species. This review extends these studies and illustrates how the microarray can also be used as an investigational tool to mine genomic data sets from strains. Three case studies describing the use of the microarray are shown and include: (1) the determination of allelic differences among *Cronobacter sakazakii* strains possessing the virulence plasmid pESA3; (2) mining of malonate and myo-inositol alleles among subspecies of *Cronobacter dublinensis* strains to determine subspecies identity; and (3) lastly using the microarray to demonstrate sequence divergence and phylogenetic relatedness trends for 13 outer-membrane protein alleles among 240 *Cronobacter* and phylogenetically-related strains. The goal of this review is to describe microarrays as a robust tool for genomics research of this assorted and important genus, a criterion toward the development of future preventative measures to eliminate this foodborne pathogen from the global food supply.

## 1. Introduction

*Cronobacter* spp. are opportunistic foodborne pathogens that are gaining attention for their ability to cause meningitis, septicemia, necrotizing enterocolitis and pneumonia in neonates (defined here as infants 28 days old or younger) and older infants [1,2,3,4]. Infantile infections have been epidemiologically linked to the consumption of contaminated batches of temperature-abused and reconstituted powdered infant formula (PIF). It is clear at this time that contamination can occur intrinsically and extrinsically. However, it has also been noted by Jason [5] that 8% (7 out of 82) of infected infants with invasive disease (defined as a culture-positive, confirmed case of septicemia or meningitis) did consume breast milk exclusively (without supplementation with PIF or powdered human milk fortifiers) prior to the onset of illness. Because PIF is not manufactured as a sterile product, it poses a significant risk if it is prepared and handled inappropriately. The most likely cause of some infections has been poor hygiene of PIF preparers. However, little is known about the source of infections involving adult cases [1]. Even though there is an abundance of published genomes for members of this genus, genomics-based epidemiology is not well documented. Primarily this is because phenotypic assessment alone cannot identify isolates at the species level; molecular assays are needed [1,2,3]. Additionally, *Cronobacter* spp. are recognized to be considerably more globally and ecologically widespread than once thought, and they have been found associated with other low water activity foods besides PIFs and follow-up formulas such as milk protein products, cereals, cheeses, licorice, candies, spices, teas, nuts, herbs, ready-to-eat foods such as pastas and vegetables, as well as filth and stable flies, and PIF or milk powder production facilities and household environments, and water [6,7]. *Cronobacter* can also survive growth conditions of extreme desiccation (high osmotic stress) and it is thought that this property influences its environmental persistence in powdered infant formula factories, other dried products and dry environments [6,7]. Together, it is now thought that this widespread occurrence and resistance to desiccation allows for the commercial re-distribution of contaminated low water activity–type foods, posing a greater risk to susceptible consumers and widening the scope of public health concerns [6,7,8,9,10,11].

Neonates are extremely vulnerable to invasive infection with *Cronobacter*, and such infections often times progress to chronic neurological sequelae such as hydrocephalus, permanent neurological damage and mental disabilities, which result in life-long developmental challenges or death; reported estimated mortality rates for this age group are as high as 80% [5,12]. *Cronobacter* has also been known to cause disease in adults, most notably in elderly and immuno-compromised individuals [4,13,14,15]. Recent surveillance data from the USA suggest that there are actually a higher percentage of *Cronobacter* infections in adults than in infants, even though invasive disease and higher mortalities are found in infants. Adults usually present clinically with extra-intestinal infections such as urinary tract and wound infections, as well as cases of septicemia and pneumonia [4].

Epidemiologically, *C. sakazakii*, *Cronobacter malonaticus*, and *Cronobacter turicensis* are the most important pathogenic species which cause the majority of severe illnesses in all age groups [2,3]. Some studies suggest that *C. malonaticus* may be responsible for more infections associated with adults than *C. sakazakii* or *C. turicensis* [15], but detailed epidemiology studies involving correctly identified organisms are warranted. Other species of *Cronobacter* associated with human illness include *Cronobacter universalis*, *Cronobacter muytjensii*, and *C. dublinensis.* Only *Cronobacter condimenti* has not been found associated with human illness, but at this time there is only one strain known [16,17].

## 2. Previous Work in the Development of a DNA Microarray for *Cronobacter* Species Identity

**Figure 1:**
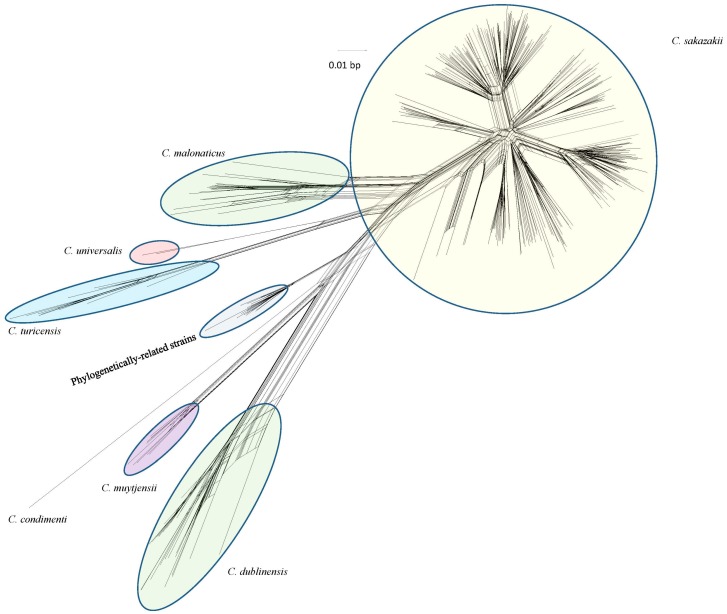
Phylogenetic analysis of 379 *Cronobacter* strains after interrogation with the Food and Drug Administration *Cronobacter* microarray. The tree was developed using the Neighbor net (SplitsTree4) joining method which was constructed using gene differences observed among the strains as determined by evaluation of the strains with the 21,658 probe sets associated with the microarray. Microarray analysis of these strains was able to group the strains into eight clusters which aligned and complemented each of the seven *Cronobacter* species epithets and phylogenetically-related species. Each species identity, originally found by using species-specific PCR assays as described by Tall et al. [18], was in complete agreement with the species identification found by microarray analysis. The scale bar represents a 0.01 base substitution (bp) per site.

Globally, the Food and Drug Administration (FDA) and its food safety collaborators share a responsibility to protect public health, and their collective capability is enhanced through the advancement of methods that can rapidly identify and characterize foodborne pathogens [1,2,3]. The rapidity of this response ensures that fewer consumers will come in contact with contaminated products and in doing so may prevent larger outbreaks from occurring [1,2,3]. The goals of this review are to describe the phylogenetic diversity among each *Cronobacter* species while also illustrating the utility of a custom-designed pan-genomic microarray platform to mine datasets for public health laboratorian use and source attribution. The sequences used to design the microarray were obtained through the whole genome sequencing efforts of a five-member International *Cronobacter* Consortium (ICC) [18]. The microarray is an Affymetrix MyGeneChip Custom Array (Affymetrix, Santa Clara, CA, USA; design number: FDACRONOa520845F) which utilized whole genome sequences and assemblies of 15 *Cronobacter* strains, as well as 18 plasmids (Table 1). Previously, a detailed pan-genome analysis of the species type strains also contributed to the design of the microarray [19]. For greater details of the design of the microarray, please see Tall et al. [18]. In total, 21,658 (19,287 *Cronobacter*-specific and 2371 virulence factor gene sequences) gene targets were used in designing the probe sets for the microarray and they followed similar schemes developed earlier by the FDA as described for *Salmonella* [20], *Escherichia coli* [21] and *Listeria* [22]. The pan-genomic DNA microarray with its concise annotation showed that it could differentiate each *Cronobacter* species and correctly identify and characterize the phylogenetic relatedness among strains isolated during surveillance and outbreak investigations. Figure 1 is an example of a phylogenetic analysis describing the evolutionary relationship of 379 *Cronobacter* strains isolated from clinical, food and environmental sources using the microarray gene difference calls [18]. In addition to distinguishing the seven *Cronobacter* species from one another and from non-*Cronobacter* species, the microarray could also group isolates into unique clusters based on their genomic diversity or allelic gene sequence divergence. Supplementary Table 1 presents the Pearson’s correlation coefficients of each summarized microarray experiment for the 379 strains shown in Figure 1. Supplementary Table S2 presents the metadata associated with these strains.

## 3. Recent Work Describing the Use of the *Cronobacter* Microarray to Data Mine Specific Alleles

The data shown in Figure 1 support and complement the phylogenic sequence divergence of the genus as described by others [18,19] and Stephan et al. [23], who used phylogenetic analyses using whole genome sequencing. Microarray analysis clearly captures and emphasizes the genomic diversity among each species member of the genus. Recently, Yan et al. [24] utilized this microarray to characterize the phylogenetic relatedness among *Cronobacter* strains obtained during an environmental surveillance study of several European PIF production facilities. Results from this study showed that the microarray was able to accurately assess each strain’s identity, could differentiate *Cronobacter* species from their nearest neighbors, and it further defined two phylogenetic lineages among the *C. sakazakii* sequence type (ST) ST-4 strains. Microarray analysis separated these groups of *C. sakazakii* isolates into multiple clusters which were also segregated according to sequence type. Interestingly, the microarray grouped 25 ST-4 isolates into two distinct subclusters. Strains from lineage 1 differed from those in lineage 2 by 24–71 genes, seven of which were phage-related and 17 were associated with the pESA3-harbored type 6 secretion system (T6SS) gene cluster. Using PCR assays described by Franco et al. [25], Yan et al. [24] were able to confirm that these unique ST-4 lineages were segregated according to differences ascribing to the strain’s type 6 secretion system which is found on the common virulence plasmid pESA3. This illustrates that the microarray improved and more accurately defined the phylogenetic genomic content of genes associated with pESA3 found in this important meningitis-causing group [25]. Together, these examples emphasize the many attributes of the *Cronobacter* microarray, the most powerful of which is its ability to assess the dispensable genome within and among each of the *Cronobacter* species. This enables one to further elucidate the evolutionary associations among disproportionately dispersed but vertically obtained genomic features, as well as horizontally acquired mobile elements.

Previously, Iversen et al. [16] showed that of the three subspecies (subsp.) of *C. dublinensis*, *C. dublinensis* subsp. *dublinensis* can utilize both malonate and *myo*-inositol, while *C. dublinensis* ssp. *lactaridi* utilizes only *myo*-inositol, and *C. dublinensis* subsp. *lausannensis* cannot utilize either of the two substrates. Grim et al. [19] showed that the alleles represented on the microarray for malonate utilization were associated with genome region (GR) 34 and the alleles for *myo*-inositol utilization were located in GR29. Microarray analysis of the species type strain *C*. *dublinensis* subsp. *dublinensis* LMG23823^T^ (synonyms: CFS237 or E187) confirmed that this strain possessed genes associated with both malonate and *myo*-inositol utilization operons, which augments and confirms the molecular presence of these operons involved in the phenotype described by Iversen et al. [16] for *C. dublinensis* subsp. *dublinensis* [18]. Furthermore, Tall et al. [18] used the microarray to mine the genomic content for both sets of these genes in 18 other *C. dublinensis* strains and demonstrated that *C*. *dublinensis* subsp. *dublinensis* strains contained both sets of gene clusters and that only *C. dublinensis* subsp. *lactaridi* possessed the *myo*-inositol utilization operon. Conversely, microarray analysis of *C. dublinensis* subsp. *lausannensis* strains confirmed that these strains lacked both gene clusters.

Another example of the usefulness of the microarray to mine phylogenetically-related information is demonstrated by performing the microarray analysis on 240 *Cronobacter* and phylogenetically-related species to determine sequence divergence among outer-membrane protein (OMP) genes which encode for several OMPs such as OmpA; OmpX; porins OmpC, D, E, and F; a conjugative plasmid transfer protein (CTP); molecular chaperon GroEL; and an OM autotransporter protein (Omatp) [26]. The microarray contains 58 OMP alleles (probe sets) representing these genes among the seven *Cronobacter* species (summarized in Supplementary Table S3). Microarray analysis demonstrated that particular alleles such as the *omatp* gene (NCBI Reference number: ABU77334) from *C. sakazakii* present on the microarray are more species-specific in that all 204 *C. sakazakii* strains and only one of 12 *C. turicensis* strains were positive for *omatp*, while representative strains of the other five species were microarray-negative. A similar sequence divergence trend was observed for *ompC* (NCBI Reference number: ABU76243) and *ompF* (NCBI Reference number: ABU77659) from *C. malonaticus* and *C. turicensis* where only seven of nine *C. malonaticus* and two of 12 *C. turicensis* strains were positive for *ompC* and *ompF*, respectively, while representative strains of the other species were negative for these species-specific OMP probe sets [27]. However, the *ompC* allele (NCBI Reference number: ABU76243) from *C. muytjensii* was determined to be present in approximately 99% (239 out of 240) of the strains. A similar trend was also seen with the *ompA* allele (NCBI Reference number: ABU79362) from *C. turicensis* which was found to be present in 99% of the strains, whereas the *ompA* allele (NCBI Reference number: ABU76166) from *C. dublinensis* subsp. *lausannensis* was only found in the *C. dublinensis* and the *C. universalis* strains. The probe set for the *groEL* allele (NCBI Reference number: ABU75458) from *C. turicensis* captured 99% of the strains analyzed except for *C. condimenti*, demonstrating that this allele is common among six of the seven species. Interestingly, the *ctp* probe set (NCBI Reference number: ABU77334) from *C. sakazakii* was also present in a high percentage of strains (96%) from all species except for *C. condimenti*, whereas the *mipA* probe sets from *C. dublinensis* subsp. *lausannensis* and *C. condimenti* (NCBI Reference number: ABU77421) only were positive in *C. dublinensis* and *C. condimenti* strains and *mipA* was not found in any other strain (species). These results again support the phylogenetic relationships generated using whole genome sequence information [19,23], and demonstrate the utility of the microarray to study the phylogenetic relationship of a specific set of alleles [11,18,19]. Outer-membrane protein genes have been submitted to NCBI under a *Cronobacter* GenomeTrakr Project: FDA-Center for Food Safety and Applied Nutrition Bioproject 258403 [27,26].

## 4. Future Directions

Important questions concerning the phylogenetic relatedness of *Cronobacter* isolates belonging to the seven species groups are just now “coming of age”, as several groups are trying to link genotype with pathotype [28,29]. Next-generation sequencing (NGS) techniques such as whole genome sequencing and microarray efforts have unambiguously spurred on *in silico* analyses of this important group of foodborne pathogens [19,30,31,32,33]. A substantial amount of genomic plasticity has been found among *Cronobacter* species [19,30,31,32,33]. For example, Zeng et al. [34] have estimated that over 16% of the genome in *C. sakazakii* strain ATCC BAA-894 consists of a prophage sequence and Grim et al. [19] described the presence of multiple T6SS systems, transposons, insertion sequence elements, and integrative and conjugative elements among the various *Cronobacter* species genomes. Studies have also shown that each species possesses a single chromosome, but may possess multiple plasmids. At least one of these plasmids, pESA3-like, possesses a common backbone consisting of the origin of the replication gene *repA* and two iron acquisition systems [19] and, according to Eshwar et al. [28], is important in virulence. These data have also shown that the seven species within *Cronobacter* can be divided into two major clades that appeared to have diverged from a common ancestor [19]. Interestingly, each *Cronobacter* species may have subsequently evolved under different selection pressures that resulted in the presence or absence of selected regions of the respective genomes. High-throughput techniques such as DNA sequencing and microarray provide deep and enriched datasets feeding to bioinformatic analysis which could provide ample insights into the emergent and unrecognized pathogenic properties of *Cronobacter* spp. Next generation sequencing also provides opportunities to conduct microbial ecological studies on this organism found in clinical, food, industry, and environmental samples, to obtain a better understanding of its interaction with hosts and foods, and thus, its survival in diverse ecological niches.

## 5. Conclusions

The principal focus of the research described in this review centers on using a pan-genome-based DNA microarray to subtype *Cronobacter* strains and to understand the sequence divergence of species-specific alleles. In this context, the microarray will also be just as useful in understanding gene expression or transcriptomics, i.e., understanding the physiology of an organism cultured under specific growth conditions. Using transcriptomics in conjunction with RNA sequencing should provide researchers with valuable insights into the survival strategies used by *Cronobacter* in powdered infant formula and other dry foods. Additional uses of microarrays would be the interrogation of strains found associated with clinical samples, which will help confirm or identify relevant virulence factors, and such microarrays would also be a useful tool to understand the distribution and prevalence of genes among species and strains. In summary, the FDA *Cronobacter* microarray is an undeniably powerful tool in the future of food safety and public health against the threat of foodborne illness epidemics caused by *Cronobacter.* Over the last few decades, *Cronobacter* contamination of commercial powdered infant formula products has apparently been reduced, but as illustrated by Patrick et al. [4], the occurrence of infections is still an ongoing problem. Furthermore, Farmer recommends that *Cronobacter* infections be made a reportable disease to the National Notifiable Diseases Surveillance System and he strongly argues that this action be ratified by the Council of State and Territorial Epidemiologists, as is the case for many other serious infectious diseases [35]. 

## Figures and Tables

**Table 1:** Number of gene features associated with the *Cronobacter* genomes that were used in the design of the microarray ^a^.

Species and Strain	Number of Alleles from Each Strain	Number Present (% Present) ^b^	Genome NCBI ^c^ Accession Numbers	Plasmid Name, NCBI Accession Numbers
***C. sakazakii* ATCC BAA-894**	2035	1904 (93.5)	NC_009778	pESA3, NC_009780.1; pESA2, NC_009779.1
**4.01C**	139	132 (94.9)	AJLB00000000.1	pESA3-like, AJLB00000000.1
**2151**	201	171 (85.0)	AJKT01000000.1	pESA3- and pCSA2151, AJKT01000000.1
**Es35**	202	188 (93.0)	AJLC00000000.1	pESA3- and pCTU3-like, AJLC00000000.1)
**Es764**	304	266 (87.5)	AJLA00000000.1	pESA3-like, AJLA00000000.1
***C. turicensis* LMG23827^T^**	4402	4039 (91.7)	NC_013282.2	pCTU1, NC_013283.1; pCTU2, NC_013284.1; pCTU3, NC_013285
***C. malonaticus***				
**LMG23826^T^**	1582	1434 (90.6)	AJKV01000000.1	pCMA1, NZ_CP013941.1/CP013941.1; pCMA2, NZ_CP013942.1/CP013942.1
**CDC2193-01**	257		JXTD00000000.1	pCTU1-like, JXTD00000000.1
***C. dublinensis***				
***C. dublinensis* subsp.^d^*dublinensis* LMG23823^T^**	781	745 (95.4)	AJKZ01000000.1	pCTU1-like, AJKZ01000000.1
***C. dublinensis* subsp. *lausannensis* LMG23824^T^**	2580	2386 (92.5)	AJKY01000000.1	pCTU1-like, AJKY01000000.1
***C. muytjensii* ATCC 51329^T^**	1754	1708 (97.3)	AJKU01000000.1	No Plasmid
***C. universalis* NCTC9529^T^**	1315	1201 (91.3)	AJKW01000000.1	pEAS3-like, AJKW01000000.1
***C. condimenti* LMG26250^T^**	2611	2498 (95.7)	CAKW00000000.1	pCTU1-like, CAKW00000000.1

^a^ Table was adapted from Table 1 as reported by Tall et al. [18]. Note that two genomes of *C. sakazakii* strains E899 and SP291 were compared with that of *Cronobacter sakazakii* strain BAA-894 and their average nucleotide identities were similar to that of strain BAA-894; ^b^ See Tall et al. [18] for more details. ^c^ NCBI: National Center for Biotechnology Information; ^d^ subsp.: subspecies

## Supplementary Materials

The following are available online at www.mdpi.com/2076-3905/6/1/6/s1. Table S1: Pearson correlation coefficients, Table S2: Metadata for tested strains, Table S3: Outer-membrane proteins (OMP) probe sets.
